# A Recent Insight into Research Pertaining to Collagen-Based Hydrogels as Dressings for Chronic Skin Wounds

**DOI:** 10.3390/gels11070527

**Published:** 2025-07-08

**Authors:** Andreea Mariana Negrescu, Anisoara Cimpean

**Affiliations:** 1 Department of Biochemistry and Molecular Biology, Faculty of Biology, University of Bucharest, 91-95 Spl. Independentei, 050095 Bucharest, Romania; andreea-mariana.negrescu@bio.unibuc.ro; 2 Research Institute of the University of Bucharest—ICUB, University of Bucharest, 050657 Bucharest, Romania

**Keywords:** collagen-based hydrogels, wound healing, skin, wound dressings, drug delivery systems

## Abstract

Affecting millions of individuals each year, chronic wounds place a substantial strain on both the healthcare system and healthcare providers, becoming a global health issue that requires a rapid and efficient solution. Unlike acute wounds that heal naturally without any external intervention, chronic wounds necessitate proper medical treatment in order to promote the wound-healing process and avoid any arising complications. However, the traditional therapeutic strategies are often limited when it comes to treating chronic wounds, which is why new approaches that facilitate the timely and effective healing of skin have been explored. Due to their unique properties, collagen-based hydrogels have been widely investigated as potential candidates for the management of chronic skin wounds, owing to their good biocompatibility, high water retention capacity, which provides a moist microenvironment, and capacity to promote cell adhesion, proliferation, migration, and differentiation for optimal tissue repair. In this context, the current paper discusses the recent advancements in collagen-based hydrogels as wound dressings, thus highlighting their potential as a future therapeutic approach for skin chronic wound care.

## 1. Introduction

As the largest organ of the human body, the skin serves as the primary physical barrier against various external invading pathogens, whilst also performing a plethora of vital functions, such as internal homeostasis regulation, excretion, external stimuli perception, and immune regulation [1,2]. Positioned as the body’s outermost layer, the skin is more susceptible to a wide range of environmental factors, i.e., physical, biological, and chemical, that can compromise its integrity. Thus, when damaged, a decline in the individual’s overall health may occur, highlighting the critical importance of maintaining the skin’s structural and functional integrity [3]. In response to injury, the outermost layer of the skin, namely the epidermis, can initiate self-healing processes and regenerate normal skin structures, primarily due to the presence of resident stem cells [4]. However, once the extent of the lesion surpasses the endogenous regenerative capacity of the skin, as seen in extensive full-thickness wounds or in individuals with a compromised innate regenerative capacity, the healing process becomes inadequate and, without proper treatment, such injuries end up closing with great difficulty and may even aggravate, mainly due to infection and other associated complications [4,5].

With a high and constant increasing incidence, chronic wounds patients are estimated to account for approximatively 2–6% of the global population, with an average annual treatment cost that exceeds 20 billion dollars only in the United States alone [6], turning these cutaneous wounds into a major clinical problem that require an urgent solving.

The use of cutaneous dressings is of the essence in wound healing, since they act as a temporary skin replacement, offering the injury protection against the external environment and bacterial infection whilst stopping the bleeding and absorbing the wound exudate [7,8]. However, the conventional dressings lack the essential anti-inflammatory and antibacterial properties required for optimal wound healing. Additionally, they may adhere to the injury site and even impede the regenerative process [7]. In this light, it is imperative that novel, multifunctional wound dressings are designed to accommodate all of the requirements for skin wound healing. In recent years, several important characteristics for an ideal wound dressing have been recognized: favourable biocompatibility with low immunogenicity and non-toxicity; good mechanical and physical durability and structural integrity; maintenance of a humid environment by long-lasting moisture preservation; excellent antibacterial properties; non-adherence to the wound site; ability to promote cell adhesion, proliferation and differentiation for an optimal cutaneous regeneration [9,10]. Encouraged by the “wet wound healing” theory, compared to other dressings, hydrogels possess a significant advantage in the biomedical field, mainly due to their high water content which provides a moist three-dimensional microenvironment suitable not only for the cellular proliferation and migration, but also for the fast transfer and exchange of metabolites and nutrients, necessary for a rapid wound repair [11,12]. Moreover, in addition to their advantageous swelling capacity, hydrogels exhibit favourable biocompatibility and adjustable physicochemical features and can be moulded into a varied selection of shapes and sizes as required [13]. Likewise, by changing their composition and structure and by loading various active molecules, hydrogel dressings can be endowed with antioxidant, antibacterial, and anti-inflammatory activities [8,14].

With the popularization of green chemistry, natural hydrogels are gradually gaining ground in front of their synthetic counterparts, mainly due to their excellent biosafety, biodegradability, and multiple biomedical applications, such as medical dressings, drug delivery systems, and regeneration scaffolds [15,16,17,18,19]. Moreover, natural polymers, such as cellulose, chitosan, collagen, and hyaluronic acid, contain bioactive endogenous factors that can speed up the natural healing process and maintain a reduced microbial status at the wound site [20]. Among these, collagen has gained significant attention as a particularly promising material for cutaneous regeneration. As a major constituent of the extracellular matrix (ECM), it is essential for preserving the structural integrity of the skin and regulating the various stages of wound healing, either in its fibrillar or soluble form [21]. Moreover, as a biomaterial, collagen exhibits excellent properties such as a good biocompatibility and biodegradability, reduced antigenicity, low toxicity, good in vivo adsorption and a superior synergism with other bioactive substances, which is why the incorporation of collagen into wound dressings has the potential to enhance the critical wound repairing process [21]. Furthermore, in the past few decades, a wide array of collagen-based wound dressings, including hydrogels, with different compositions, have been clinically approved and commercialised. One example is the Integra^®^ Dermal Regeneration Template, a membrane based on a layer of type I collagen cross-linked with chondroitin-6-sulfate and covered with a semipermeable silicone sheet used in the treatment of venous ulcers and combat-related wounds [22]. Other collagen-based dressings include FIBRACOL and CollaSorb, with a composition of 90% collagen, 10% alginate, and 90% pure collagen mixed with 10% calcium alginate, respectively; Wun’dres gel, a mixture of collagen–phenol–allantoin, Promogran with 55% collagen and 45% oxidized regenerated cellulose; and Medifil, a 100% non-hydrolyzed bovine type I-derived collagen in the natural triple-helical molecular form [23].

However, in spite of its outstanding properties and its already proven clinical efficiency as wound dressings, collagen exhibits a series of disadvantages such as thermal instability, enzymatic degradation, and mechanical strength, which can be improved by using various methods, i.e., crosslinking, blending, grafting, polymerization, and covalent conjugation [24].

In light of this, the present review aims to summarize the recent advancements in the field of wound dressings with regard to the collagen-based hydrogels for cutaneous regeneration. By critically analysing the latest research findings, i.e., processing methods and new formulations that could improve the long-term skin wound healing outcomes, and by highlighting the future directions of collagen-based hydrogels, we aim at offering researchers and clinicians who seek the optimization of wound healing outcomes, valuable insights into an ever growing and dynamic field. It is important to note that in this review, we will confine our discussion only to studies in the field that have been published over the last 5 years, with an emphasis on data reported within the last two years, in order not to overlap with previously published reviews that cover the same matter. Furthermore, although gelatin is a collagen derivative widely used in a wide array of biomedical applications, it has been intentionally excluded from the scope of this review. Gelatin is an inexpensive, natural polymer derived from the partial hydrolysis of the non-soluble collagen, sharing an identical amino acid sequence and exhibiting several functional properties similar to collagen, including biocompatibility and hydrogel-forming ability [25]. However, significant differences between the two polymers do exist, affecting the gelatin-based hydrogels’ performance as wound dressings. For instance, as a denatured form of collagen, gelatin lacks the triple helix and hierarchical organisation of native collagen, which are essential for mimicking the biological and mechanical functions of natural ECM [25]. This structural difference profoundly influences the characteristics and potential of the resulting hydrogels as wound dressings, mainly due to their inability to induce certain biological responses that native collagen is inherently programmed to elicit in cells involved in the wound healing process [26]. Moreover, compared to the collagen-based hydrogels, gelatin hydrogels tend to degrade faster and display a poorer mechanical strength due to their low stability at physiological temperatures in the absence of crosslinking [27]. These factors are critical in the context of chronic wound healing, where a sustained scaffold integrity and a favourable microenvironment are essential for promoting cellular migration, proliferation, and differentiation. For instance, Mousavi et al. [28] investigated by comparison the physical properties and biological response of the newly developed collagen/chitosan and gelatin/chitosan-based hydrogels for wound dressing applications. The results revealed that even though the swelling ratio of the collagen/chitosan hydrogel (10%) in phosphate buffer saline (PBS) after 70 h was lower than that of its gelatin/chitosan counterpart (30%), the gelatin containing hydrogel displayed a poorer scaffold integrity (1.3-fold) and a lower thermal stability. Moreover, the values of water vapour transmission rate (WVTR) for the collagen/chitosan-based hydrogel (2750 ± 436.50 g/m^2^/day) was found to be close to the widely acknowledged interval (2000–2500 g/m^2^/day) suitable for maintaining the appropriate moisture levels at the wound site. In addition, the degradation rate of the collagen/chitosan-based hydrogels (42.85 ± 15.87%) in PBS after 15 days, was slower than that of gelatin/chitosan-based hydrogels (33.82 ± 7.3%), indicating that in terms of degradation, the collagen containing formulation is better suited for wound dressing applications. Consequently, while gelatin is a popular option for hydrogel fabrication due to the production-associated benefits such as ease of processing and cost-effectiveness [29], the definite hierarchical structure of collagen confers enhanced functional properties that make collagen-based hydrogels more effective as dressings for long-term wound healing. Therefore, this review will focus exclusively on hydrogels formulated with native or minimally modified collagen sources.

The first part focuses on the natural wound healing process, the role of collagen in wound healing and the various sources of collagen, either natural or synthetic, while the second part is comprised of an overview of the collagen-based hydrogels, from methods of fabrication to in vitro and in vivo experiments based on collagen hydrogels for skin regeneration and wound healing. Lastly, the challenges, limitations, and future research directions are also discussed.

## 2. Skin and Wound Healing Process

### 2.1. The Skin Structure

**Figure 1:**
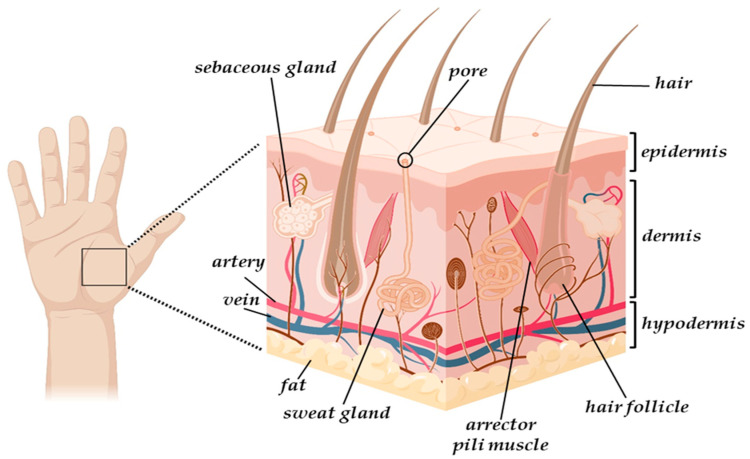
Schematic representation of skin structure. Created with BioRender.

Skin is a sophisticated organ comprised of three distinct layers: namely, the epidermis, dermis, and hypodermis (Figure 1). The outermost layer, the epidermis, is a non-vascular epithelial tissue measuring between 75 and 150 μm in thickness. While keratinocytes make up the majority of its cellular composition, it also contains melanocytes, Langerhans cells, and Merkel cells, each contributing to its diverse functions [30]. Accounting for approximately 90% of the cells in the human epidermis, the keratinocytes play a crucial role in reducing water evaporation and protecting the skin against external traumas, while the melanocytes provide protection against ultraviolet radiation through the production of melanin, which determines skin tone [30]. In addition, the Langerhans cells combat bacteria, while the Merkel cells function as tactile and endocrine cells [30]. The next skin layer is represented by the dermis, a highly vascularised thick connective tissue (2 mm to 4 mm) located between the epidermis and the subcutaneous tissue, in which more complex cell types can be found [31]. The structural integrity of the cutaneous tissue is heavily impacted by its components, i.e., fibroblasts and the ECM components such as elastin, collagen, hyaluronic acid, and glycosaminoglycans, which all together contribute to the skin’s tonicity and elasticity [31]. Moreover, it also contains blood and lymphatic vessels, receptors, macrophages, and various appendages, such as hair follicles, sebaceous and sweat glands, characteristics which allow the involvement of dermis in the vascular, sensorial, and immune systems and connective tissue [1]. Finally, the subcutaneous tissue, or hypodermis, is a well-vascularised layer composed of adipocytes and collagen, with a primary role in thermoregulation, mechanical protection, and dermal-skeletal attachment [32].

### 2.2. The Natural Wound Healing Process and Types of Skin Wounds

Due to the constant and excessive exposure of skin to various internal and external factors such as physical, mechanical, chemical and other environmental risk factors, the anatomical and functional integrity of the cutaneous tissue can be physiologically disrupted, resulting in wounds [33], which based on their healing time are commonly categorized into acute and chronic wounds [15,34,35]. Resulting from traumas that damage the integrity of skin, acute lesions are considered to be a type of wound in which the tissue’s structure and function are recovered completely through a natural self-healing process, without the intervention of any external factors [35]. Normally, small burns, minor surgery wounds and minor cuts heal within 8–12 weeks, but the healing rate of acute wounds depends on two wound characteristics, namely the size, depth and location of the wound and, most importantly, on the individual’s overall condition, i.e., age or underlying disease [36].

**Figure 2:**
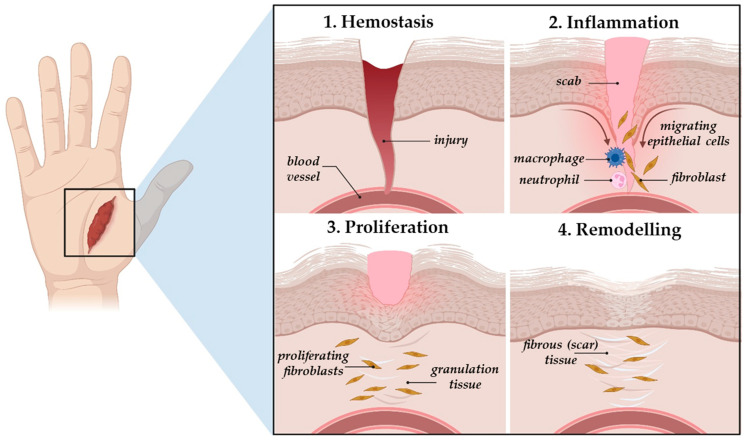
Schematic representation of the four stages of the natural wound healing process. Created with BioRender.

The physiological wound repair process is a highly regulated and intricate sequence of events that can be divided into four overlapping stages, i.e., haemostasis, inflammation, proliferation, and remodelling (Figure 2), in which each phase involves intricate, dynamic interactions that contribute to tissue repair [34]. Following injury, the wound will enter the initial stage of the healing process, namely haemostasis, in which the blood flow is restricted through both vasoconstriction and clot formation [32]. Once the blood cloth is formed, inflammatory cells (e.g., neutrophils and monocytes), under the action of cellular factors and locally secreted growth factors, will migrate to the site of injury and start clearing out any foreign bodies, bacteria, damaged endogenous tissue and reactive oxygen species (ROS) [37]. The purpose of the inflammatory stage is to prevent bacterial infection and to establish a microenvironment beneficial to the wound healing process. To achieve this, the migrating monocytes will differentiate into macrophages, which, along with the resident macrophages, will secrete a range of inflammatory mediators that help create a balanced inflammatory environment that supports tissue regeneration. Initially, in order to stimulate inflammation, the macrophages will differentiate into a classically activated M1 phenotype, secreting pro-inflammatory mediators (cytokines, chemokines), but as the process progresses, and the bacteria and dead tissue are removed, the macrophages will change their polarization state towards an anti-inflammatory phenotype (M2). The alternatively activated M2 macrophages will secrete anti-inflammatory cytokines and growth factors that help direct the process towards the next stage of wound healing, namely proliferation [34,38]. During this phase, fibroblasts, keratinocytes, and endothelial cells migrate to the injury site and start filling it, resulting in the formation of a pale pink granulation tissue [32]. Additionally, new blood vessel also forms (angiogenesis) and fibroblasts start secreting collagen and other ECM components to promote re-epithelialization. In the final stage, the myofibroblasts, which differentiated from the proliferating fibroblasts, will cover the site of injury, leading to scar formation [39]. During wound contraction, the granulation tissue will transform into the ECM, which, under the action of the matrix metalloproteinases (MMPs), will undergo a series of modifications that will lead to tissue restructuring and scar formation [39].

**Figure 3:**
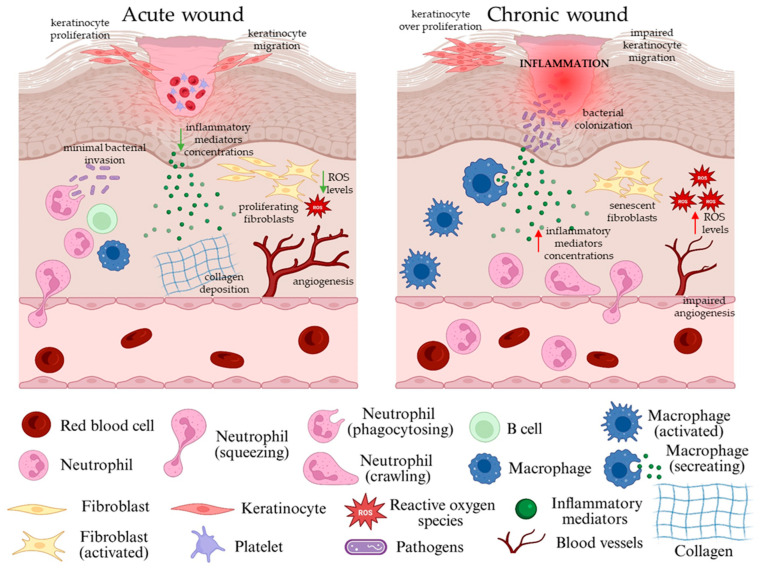
Schematic representation of the acute and chronic wound phenotype. Created with BioRender.

In healthy individuals, wound healing occurs properly and without interruption, but various pre-existing health conditions/pathologies may result in a disturbance in one or more of the aforementioned phases, leading to a slow and impaired healing process and chronic wound formation [40]. Chronic wounds are defined as wounds that fail to undergo the normal healing process or restore the structural and functional integrity within a time frame of 3 months since initial injury [40]. The formation of chronic wounds involves a highly intricate process driven by various contributing factors, starting from an impaired blood flow to peripheral vascular conditions, systemic illnesses, and infections. These wounds often lead to a significant loss of tissue that, in more advanced cases, may compromise deeper structures, including nerves, joints, and bones [40]. Compared to acute wounds, chronic wounds display a different phenotype, as presented in Figure 3.

### 2.3. The Role of Endogenous Collagen in the Natural Skin Wound Healing

As an important component of the skin matrix, collagen plays a role in each of the wound healing stages. Thus, while not produced anew in the haemostatic and inflammatory stages, the exposed collagen fibres from damaged blood vessels trigger the clotting cascade, resulting in fibrin clot formation and prevention of further blood loss [8,41]. Type I collagen and type IV collagen fragments can serve as effective chemoattractants, helping to recruit immune cells (e.g., neutrophils) to the injury site by enhancing phagocytosis and immunological responses and influencing gene expression [42]. Furthermore, data reported in literature showed that collagen can promote the polarization switch of macrophages towards an anti-inflammatory and pro-angiogenic phenotype via the activation of the microRNA signalling pathway [42]. In the proliferative phase, migrating fibroblasts begin synthesizing type III collagen, which acts as a temporary scaffold for the formation and organization of new cutaneous tissue. Moreover, research also indicates that the C-propeptide fragment of type I collagen can exert chemotactic effects on endothelial cells [42], thus promoting new blood vessel formation and tissue vascularization. As the process progresses, the collagen matrix substitutes the pre-existing fibronectin-rich matrix, and during adult wound healing, type I collagen replaces type III collagen, leading in the end to the formation of the scarring tissue [33].

## 3. Collagen Structure and Sources

### 3.1. Collagen Structure

**Figure 4:**
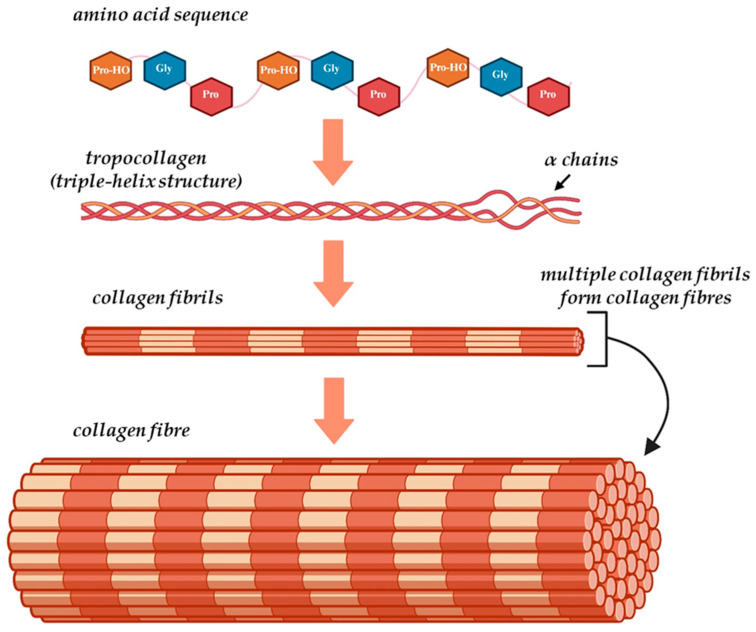
Schematic representation of collagen synthesis and structure. Created in BioRender.

Accounting for almost one-third of the total protein mass in the human body, collagen is one of the most important ECM components, being responsible for the matrix’s durability and elasticity [43]. There are over 20 types of collagen in nature, and all of them display a hierarchical structure that starts from the primary amino acid sequence and ends with their organization into complex fibres [43]. The unique structure of collagen (Figure 4) consists of three polypeptide molecules, known as “α chains,” positioned parallel to one another and coiled in a left-handed polyproline II-type helix (procollagen), that intertwine to create a right-handed triple helix structure referred to as tropocollagen [7,43,44]. The primary structure of collagen is represented by the specific sequence of amino acids in the polypeptide chains, and is characterised by the presence of a repeating glycine (Gly)-Xaa-Yaa residue triplet, in which Xaa and Yaa are usually proline (Pro) and hydroxyproline (Pro-HO) residues, respectively [45]. The repetitive presence of Gly and high content of Pro, combined with the hydrogen bonds and electrostatic interactions formed by Pro-HO between the aspartic acid and lysine residues, helps secure the compact organization of the three α chains into the tropocollagen configuration [46]. This unique conformation of the collagen triple helix, coupled with the strong bonding between the amino acids, gives collagen fibres their high flexibility and resistance to stretching [43]. The 29 various types of collagens are comprised of 25 different chains that are assembled in various combinations; while the three α chains in a collagen molecule can be identical (homotrimeric), heterotrimeric triple helices, where one or more of the α chains differ, are more common than homotrimeric ones [44].

### 3.2. Collagen Sources for Hydrogel Manufacturing as Skin Wound Dressings

Currently, collagen can be extracted from a varied selection of sources, including natural (e.g., animal and plant) sources, or it can be obtained through the use of recombinant protein production systems based on bacteria, yeast, insects, artificial fibrils, plants, or mammalian cells [23,47]. Among the aforementioned sources, animal-derived collagen, typically from avian, bovine, or porcine origins, remains the primary material used in collagen-based dressings recommended for full- or partial-thickness wounds with minimal or moderate exudate [48]. However, these animal-derived collagen dressings are unsuitable for patients with third-degree burns or dietary restrictions, either imposed by allergies to various animal products or by religious and cultural beliefs [49]. Furthermore, the potential risk of zoonotic disease transmission, ethical objections regarding to the use of animal-derived products, and environmental concerns related to the long-term sustainability and impact of animal agriculture [50], further underscores the necessity of identifying new collagen sources that are not only suitable for skin wound applications but also can overcome the limitations associated with animal-derived sources.

As a biomaterial with a wide range of sources, marine-derived collagen has garnered increasing scientific attention, mainly due to its unique properties such as excellent biocompatibility, low immunogenicity, high degree of bio-adhesion, and good biodegradability [49,51]. In addition to its biological advantages, the use of marine-derived collagens significantly reduces the probability of disease transmission, lowers the production costs, and improves the collagen yield, whilst contributing to more sustainable practices by minimizing waste from collagen-rich marine by-products [52]. The skin, scales, fins and bones of fish like salmon, pollock and cod are abundant in collagen, which in contrast to the animal-derived collagen, when subjected to chemical or enzymatic hydrolysis, it forms smaller sized collagen peptides with a low molecular weight, a higher hydrophilicity and a more effective body adsorption [43,53]. In a study by Li et al. [54], the potential of a novel composite hydrogel based on Tilapia-skin-derived collagen and alginate as an efficient wound dressing for refractory wounds was explored. Through both in vitro and in vivo studies, the hydrogel was shown to be non-cytotoxic, with the cell survival ratio of the NIH-3T3 fibroblasts exceeding 85% by the third day in culture. The in vivo findings further confirmed the wound healing efficacy of the newly developed composite hydrogel, revealing an accelerated healing rate (*p* < 0.01) and a reduced scar area after 21 days compared to the collagen-only control group. Similarly, in the quest for an ideal wound healing dressing, Akter et al. [55] designed and investigated a more complex gel formulation based on Tilapia-skin collagen with human amniotic membrane (AM) and *Centella asiatica* extract. After 21 days of treatment, the composite gel showed a superior wound healing ability by significantly accelerating wound contraction, from 55.32 ± 2.67% (observed in the case of the control group) to 95.75 ± 0.44%. Moreover, the wound epithelialization analysis revealed a considerably higher degree of epithelialization in the case of the wounds treated with the composite collagen (23.67 ± 0.763 days) as opposed to the untreated control group. Echoing these results, Baydogan et al. [56] further demonstrated the clinical feasibility of an alginate/Tilapia skin collagen-based hydrogel in skin burn wound treatment. Their findings pointed to an increase in collagen production and a notable modulation of MMP-2 and MMP-9 enzyme levels, both of which play pivotal roles in ECM remodelling during the natural wound healing process. Marine collagen peptides isolated from different species also hold great promise in the field of skin wound regeneration, as previously reported by Yang et al. [53]. Through the in vitro scratch assay, it was shown that marine peptides extracted from *Nibea japonica* were capable of increasing the closure rate and promote the migration of the NIH-3T3 cells in a dose-dependent manner–the highest wound closure rate (100% after 24 h) was attributed to the highest concentration (50 μg/mL) of marine peptides. Additionally, these peptides also increased the in vitro levels of certain growth factors associated with the natural wound healing process, such as epidermal growth factor (EGF), fibroblast growth factor (FGF), vascular endothelial growth factor (VEGF) and transforming growth factor (TGF-β), suggesting a mechanism of action closely linked to the NF-κB signalling pathway. Due to its good antioxidant activity that could potentially attenuate the excessive inflammatory response, marine snail peptide may represent an effective strategy in the hydrogel manufacturing for skin wound applications [57]. Using the marine snail peptide YIAEDAER, alongside carboxymethyl chitosan and carboymethyl cellulose, Ren et al. [57] fabricated a novel hydrogel designed to be injectable and self-healing. Their findings showed that the newly developed composite hydrogel displayed a remarkable biocompatibility (97% viability), a potent antioxidant activity [DPPH (2,2-diphenyl-1-picrylhydrazyl) scavenging rate of 33% vs. 19% in the control group], and effective antimicrobial properties. Moreover, after 14 days, the treated wounds exhibited a drastic reduction in wound area (2%) compared to 24% in the untreated controls. Immunofluorescence staining revealed enhanced angiogenesis—indicated by upregulated CD31 [PECAM-1 (Platelet Endothelial Cell Adhesion Molecule-1) and Alpha Smooth Muscle Actin (α-SMA) expression—and a favourable inflammatory profile, marked by increased interleukin (IL)-10 and suppressed IL-6 levels. Altogether, these studies highlight a growing body of evidence supporting the use of marine-derived collagens in wound care. Whether through accelerating cellular proliferation, modulating the immune response, or enhancing vascularization, these hydrogels represent a promising shift toward more effective and biologically attuned wound healing strategies.

Although the isolation of collagen from natural origins represents one of the easiest and frequently used approaches to obtain collagen, it is not without its limitations. For example, most of the time, the collagen structure can be affected during the preparation process, or some of the rarest collagen types cannot be efficiently isolated. Moreover, as mentioned above, the isolated collagen may elicit pathogenic and immune adverse reactions in some individuals, while the high prevalence of diseases and health-related conditions in animals can limit the use of animal raw products [8]. All these together led to a new direction in collagen production, namely recombinant collagen. The recombinant collagen is obtained by splicing gene fragments of collagen into suitable vectors using the tool enzymes, and their subsequent transfer into host cells to induce expression. By using this method, the structural sequence, quality, processability, water-solubility, and immunogenicity of the obtained collagen can be controlled and customized as needed [50]. Up to now, a wide range of recombinant expression systems have been employed with the purpose of obtaining recombinant human collagens (HRC); amongst them, bacteria (e.g., *Escherichia coli*) [58], animal cells, transgenic plants and animals, yeasts [59], etc. However, the bioengineered recombinant collagen cannot substitute animal-extracted collagens, mainly due to its limited quantity, ethical constraints, and the disregard of researchers for the impact of high-level structure on the recombinant collagen performance as opposed to smaller collagen peptide fragments [7,50].

## 4. Fabrication Methods of Collagen-Based Hydrogels

**Figure 5:**
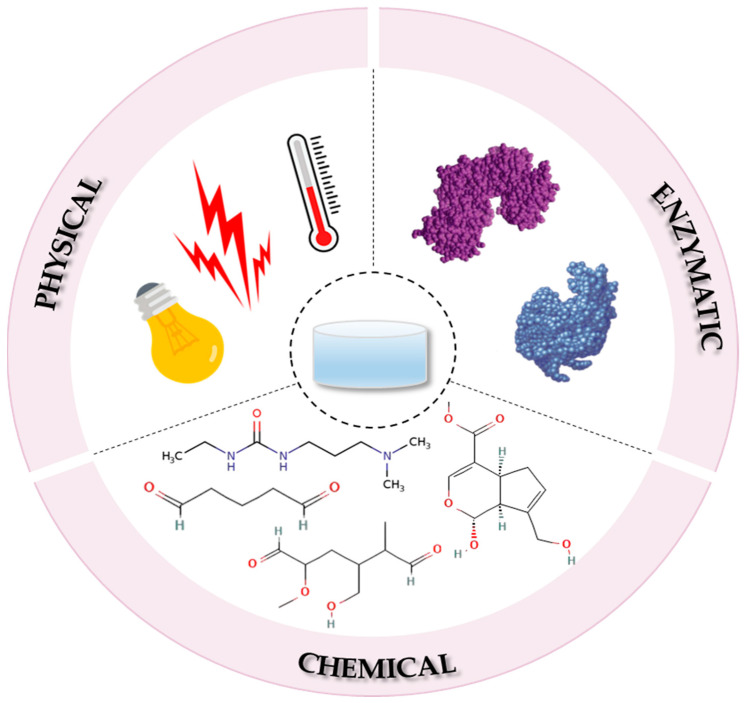
The schematic illustration of the collagen-based hydrogels’ crosslinking processes. Physical crosslinking involves approaches such as irradiation and DHT. Chemical crosslinking requires the use of agents like glutaraldehyde, genipin, dialdehyde starch, and carbodiimides. In contrast, enzymatic crosslinking employs enzymes such as transferases (e.g., glutamine transaminase), hydrolases (e.g., lysyl oxidase), and oxidoreductases (e.g., horseradish peroxidase).

Since the self-assembly behaviour of collagen is fundamental to collagen-based hydrogel processing, currently the main strategy through which hydrogels are fabricated is the crosslinking process, which based on the type of bonds formed between the polymeric chains during crosslinking, can be divided into physical, chemical and enzymatic techniques (Figure 5) [60,61].

### 4.1. The Physical Crosslinking Process

During the physical crosslinking process, the collagen molecule is cross-linked under the effect of various physical treatments such as ultraviolet (UV) light, heating, freeze-drying cycles and γ-ray exposure, which lead to the formation of a reversible three-dimensional (3D) network structure, in the form of a viscoelastic gel system [8]. Compared to the chemical crosslinking method, the resulting hydrogels lack the potential cytotoxic effect of the used chemical agents [61] and exhibit self-healing properties in the sense that, under specific conditions such as heat, they can change states from solid to liquid and re-crosslink when the external factors are withdrawn [62]. In addition, they also exhibit a high water sensitivity and thermal reversibility, with a short lifespan (from a few days to a month at most) in physiological conditions. Thus, in this form, the physically cross-linked hydrogels may serve as drug delivery platforms where the short-term release of the active substance is required [63].

The formation of cross-linked collagen through UV radiation is based on the formation of free radicals on aromatic amino acid residues, such as tyrosine and phenylalanine [64], which interact with each other and form chemical bonds between the collagen molecules [65]. Being a physical method, UV-induced crosslinking does not entail the use of any additional, potentially cytotoxic reagents. However, since collagen is sensitive to UV light, prolonged exposure and high temperatures can accelerate its photo-degradation [65]. Therefore, during UV irradiation, the crosslinking and denaturation processes oppose one another, which is why the final balance between these two may influence the overall mechanical and structural properties and degradation rate of the collagen-based hydrogels [66]. Moreover, the inability of UV irradiation alone to achieve a high degree of cross-linked fibres has prompted researchers to explore more efficient methods, which often involve the use of a photosensitizer in combination with UV light to generate both intra- and intermolecular bonds within the collagen fibres [8]. For example, the UV-riboflavin or UV-semi-synthetic gelatin methacrylate (Gel-MA)-induced crosslinking reaction of collagen is often used in treating skin wounds [67,68], while UV-A-riboflavin crosslinking reaction is commonly used to treat ocular injuries [69]. However, for all its advantages, this alternative method does have its limitations. One of the major drawbacks is the reaction threshold that arises when the rate of the crosslinking process is accelerated, which can affect the efficiency and control over the final structure [70]. It is worth mentioning that the crosslinking of collagen via UV irradiation is highly dependent on the ability of the UV light to penetrate the designed scaffold, meaning that only thin and translucent materials can be fabricated using this method [65]. Gamma rays are capable of inducing rapid and uniform crosslinking of polymers through the synergistic effect of the generated free radicals, thereby eliminating the need for chemical crosslinking agents [71]. However, unlike UV light, gamma radiation offers a significantly greater penetration depth into the designed scaffold and a more precise control over the crosslinking process [72]. Nevertheless, excessive exposure may induce collagen chain scission and denaturation, which is why, in order for the structural integrity of the polymer to be fully preserved, dose optimization should be carefully performed [72]. Among the irradiation-based crosslinking methods, electron beam (e-beam) radiation is considered in literature as the least damaging to collagen [73], primarily due to its ability to trigger rapid chemical reactions within a short amount of time without giving rise to high temperatures [73]. For instance, Riedel et al. [74] investigated how high-energy e-beam crosslinking can be employed to precisely tune collagen characteristics for diverse ECM model systems. The quantification of the 3D pore size of the collagen network highlighted a dose dependent effect that translated into a directly proportional relationship between the pore size and the crosslinking density, i.e., a higher crosslinking density correlated with a reduced pore size (50 kGy–75% reduction of the initial pore size; 100 kGy–50% reduction of the initial pore size). The in vitro examination demonstrated that both the unirradiated and irradiated hydrogels displayed a viability of approximately 95%, without significant differences between the analysed irradiation doses.

Apart from the irradiation-based techniques, physical crosslinking of collagen can also be accomplished by the use of dehydrothermal (DHT) treatment. DHT technique is a chemical reagent-free process that involves the exposure of collagen to elevated temperatures (i.e., >90 °C) under vacuum conditions. This method promotes the removal of water molecules and facilitates the formation of intramolecular amide bonds between collagen chains [75]. When applied properly, it preserves the inherent structure of collagen, thereby enhancing its stability and mechanical characteristics [76]. Tian et al. [77] designed a 1-(3-Dimethylaminopropyl)-3-ethylcarbodiimide/N-Hydroxysuccinimide-dehydrothermal (EDC/NHS-DHT) dual-cross-linked construct comprised of a silica gel, collagen membrane, and a porous collagen scaffold for enhanced full-thickness wound healing. The dual EDC/NHS-DHT cross-linked collagen composite bilayer (EDCCB) exhibited a significantly higher cross-linking degree (79.5%) compared to the DHT-treated collagen composite bilayer (DCCB, 15.5%) and the EDC/NHS-cross-linked collagen composite bilayer (19.4%). It also demonstrated superior mechanical strength (non-cross-linked composite: 30.96 kPa; DHT-cross-linked: 40.33 kPa; dual cross-linked: 50.78 kPa), excellent water uptake capacity (swelling ratio: DHT-cross-linked—72; dual cross-linked—95), and appropriate biodegradability in PBS (mass degradation: DHT-cross-linked—91.6%; dual cross-linked—40.4%). The animal studies revealed that the dual cross-linked composite promoted cutaneous regeneration through an accelerated re-epithelialization, stimulation of collagen deposition, and migration of skin appendage cells.

However, it should be noted that DHT crosslinking may cause collagen deformation due to the rearrangement of its tertiary structure into a less ordered form when exposed to elevated temperatures and prolonged treatment times. Additionally, because the crosslinking process can take several days, DHT-cross-linked collagen supports have limited biomedical applications [65].

### 4.2. The Chemical Crosslinking Process

Opposed to the physical crosslinking strategy, the chemical-induced crosslinking process requires the action of a synthetic reagent that, under conditions of heat, light, or irradiation, introduces exogenous crosslinks between and within the collagen fibres [78]. Through the presence of the covalent bonds, the hydrogel network is reinforced, becoming more resistant to environmental changes like fluctuations in pH and temperature. As a result, hydrogels cross-linked via the chemical method tend to display enhanced long-term stability and mechanical strength [63]. However, even though this method is one of the most prevalent strategies that allows the rapid formation of collagen cross-linked fibres, the utilized crosslinking agent can still remain after washing [8], and the properties of the resulting cross-linked collagen-based support are heavily influenced by the used cross-linker [79]. Moreover, the efficiency of the process is heavily influenced by temperature and the reagent’s concentration, factors that determine the stability of the cross-linker, the number of free amino acid residues that can react with the cross-linker, and the bond energy that is associated with each cross-link [61].

The main cross-linkers, such as glutaraldehyde, genipin, dialdehyde starch, and carbodiimides, will be discussed in detail below.

#### 4.2.1. Glutaraldehyde

Due to its cost efficiency and high reactivity, glutaraldehyde was the first crosslinking agent utilised in collagen-based hydrogel preparation through chemical crosslinking. However, despite its extensive use, the exact mechanism behind the glutaraldehyde-collagen interactions is not fully elucidated, but what is known is the fact that the glutaraldehyde-protein crosslinks are formed through the reaction between the aldehyde groups of the crosslinking agent with the ε-amine groups of lysine or hydroxylysine residues within the collagen molecule [8]. This primary reaction leads to the formation of an intermediary Schiff base, which in turn will cause several subsequent reactions, that in the end will result in the formation of the cross-linked collagen fibres [61]. It is important to be mentioned that depending on the concentration of collagen participating in the reaction, the mechanical properties of the resulting hydrogel will differ. Thus, if the collagen contraction is high, an inhomogeneous reaction will occur, leading to a hydrogel with undesirable properties [80]. However, despite its advantages and wide application, glutaraldehyde is cytotoxic due to the inability of the by-products and unreacted chemicals to be completely removed at the end of the crosslinking reaction [81].

#### 4.2.2. Dialdehyde Starch

Dialdehyde starch (Das), a derivative of starch, is a macromolecular aldehyde that can be obtained through the oxidation process of the natural starch with various oxidants such as sodium periodate or periodic acid [82]. Due to the presence of many active aldehyde groups, it exhibits excellent physico-chemical and biological properties, such as strong adhesion and alkaline solubility, and it can be used as a crosslinking agent, catalysing the reaction between the amino and imino groups of collagen [83]. These interactions alter the network structure of the collagen, leading to modifications in the mechanical properties of the resulting hydrogels. For instance, Valipour et al. [84] designed collagen (Col) (4% *w*/*v*)/chitosan (Ch) (2% *w*/*v*) based hydrogels, cross-linked with varying amounts of Das (2% *w*/*v*), specifically 0.5, 1.0 and 1.5 mL, as potential candidates for chronic wound therapy, and as expected, the introduction of the cross-lining agent led to modifications in the mechanical properties of the hydrogels, dependent on the used Das concentration. Among the formulations, the Col/Ch/Das_0.5_ hydrogel exhibited a superior swelling capacity after 60 min in PBS (128.00 ± 1.36) and a higher biodegradability rate after 23 days in simulated body fluid (SBF) (50.16%) compared to the other two hydrogels, Col/Ch/Das_1_ (88.41 ± 5.60 and 39.18%, respectively) and Col/Ch/Das_1.5_ (74.04 ± 0.36 and 41.36%, respectively) (*p* < 0.05). The significantly higher swelling capacity of the Col/Ch/Das_0.5_ hydrogel, compared to the formulations with higher Das content, can be attributed to reduced structural tightening at lower Das concentrations. This effect arises from an increase in the number of available crosslinking sites at higher Das concentrations, which leads to the formation of a denser and stronger network with a diminished water absorption capacity [85]. Moreover, WVTR exhibited a decreasing trend with increasing Das content, from 51.78% in Col/Ch/Das_0.5_ to 44.33% in Col/Ch/Das_1_, and further down to 40.01% in Col/Ch/Das_1.5_. These results can be explained by the fact that higher concentrations of Das create a denser network with reduced porosity, which in turn affects the water vapour diffusion. Nevertheless, these values remain within the range considered sufficient to maintain an optimal moisture level at the wound site, as suggested by Queen et al. [86]. In addition, scanning electron microscopy (SEM) showed that varying Das concentrations influenced the porosity of the hydrogels, with the lowest Das concentration resulting in the most favourable pore size for wound healing applications. As the degree of cross-linking increases, the intermolecular bonding is enhanced, which consequently leads to a reduction in both size and volume of the pores. Notably, collagen-based hydrogels with larger pores exhibit a wide selection of benefits for an optimal wound healing process, including improved cell infiltration, efficient nutrient and drug delivery, and expedited removal of metabolic wastes. Thus, the modulation of the Das content enables a precise control over the physicochemical properties of collagen-based hydrogels, namely, swelling behaviour, mechanical strength, and pore size, through alterations in their hydrophilicity and microstructural heterogeneity. Moreover, dialdehyde starch is non-toxic, biodegradable, and exhibits antiviral activity, making it a sought-after candidate in the preparation of collagen-based hydrogels [87].

#### 4.2.3. Carbodiimides

Less toxic to cells than glutaraldehyde, 1-ethyl-3-(3-dimethyl aminopropyl) carbodiimide (EDC), often used in combination with N-hydroxysuccinimide (NHS), is a good example of a commonly used carbodiimides that belongs to the unique class of zero-length cross-linkers–special chemical reagents that enables the covalent bonding of two polymeric chains without any additional atoms or linkers in between them, effectively creating a direct bond [8]. These crosslinking agents facilitate the intermolecular binding between the amino and the carboxyl groups of the glutamic or aspartic acid residues, with a highest level of efficiency in moderately acidic conditions, i.e., pH of approximately 4.5. However, the crosslinking reaction can also occur in physiological conditions, i.e., 37 °C, pH 7.4, turning this crosslinking approach into an attractive option for various biomedical applications [61]. Moreover, during the crosslinking process, the carbodiimides are converted into non-toxic water-soluble urea derivatives that can be washed away with ease at the end of the crosslinking process [88], minimizing the risk of the by-product release into the collagen matrix. Therefore, carbodiimides exhibit a reduced cytotoxic potential while significantly enhancing the physicochemical properties of the collagen-based hydrogel [89], resulting in hydrogels that support cell adhesion and proliferation. Liu et al. [90] manufactured an EDC/NSH cross-linked polyacrylamide (PAM)-collagen-based hydrogel as a potential material for various biomedical applications, including skin wound healing. When compared to the traditional collagen-based hydrogels, the hybrid system displayed an adjustable stiffness, high transparency and hydrophilicity, and excellent biocompatibility. Thus, by varying the concentrations of acrylamide or collagen, the newly formulated gels exhibited an adjustable tensile modulus from 3.2. kPa to 392.5 kPa and a compressive modulus starting from 1.2 kPa to 54.0 kPa, both observed when the collagen concentration was fixed at 6% and the acrylamide increased from 7% to 20%. Furthermore, in the absence of a crosslinking agent, the resulting hydrogel presented an irregular and non-uniform porous structure, whereas the EDC/NHS treatment produced a more uniform and compact porous architecture within the hydrogel network. Follow-up studies, including in vitro cell assays, cryopreserved embryo cultures, and animal experiments, confirmed the hydrogel’s effectiveness in supporting cell proliferation across a range of stiffness levels. In another study, Sionkowska et al. [91] explored the effects of crosslinking conditions on the properties of marine-derived collagen films, using EDC and NHS as crosslinking agents. The evaluation of the tested collagen films indicated that the sample prepared by adding the EDC directly to the fish-derived collagen solution exhibited the lowest Young’s modulus (approx. 5-fold), tensile strength (approx. 2-fold), and breaking force parameters (approx. 1.5-fold), and the highest elongation at break (approx. 3-fold). Moreover, differences in the swelling degree and durability of the developed films were also observed, i.e., the immersion-cross-linked films displayed the lowest swelling rates (Coll_EDC_d and Coll_EDC/NSH_d–146 ± 24 and 191 ± 40 vs. Coll_EDC/NSH and Coll_EDC–945 ± 151 and 0; as measured in PBS after 4 h) and were more durable than their counterparts (Coll_EDC and Coll_EDC/NSH films disintegrated after 8 h). In terms of hydrophilicity, the addition of the crosslinking agents significantly reduced the films’ hydrophilic nature (contact angle: 70.8°–native collagen; 87.7° Coll-EDC film; as measured in glycerin) and implicitly their wettability. These findings demonstrate that EDC/NSH crosslinking can significantly improve the mechanical strength, structural uniformity, and durability of collagen-based hydrogels and films, while also allowing fine control over their biocompatibility and stiffness, at a compromise in hydrophilicity and wettability. All together, they highlight the relevance of EDC/NHS systems in chronic wound care, where both structural stability and bioactivity are required.

#### 4.2.4. Genipin

Alongside synthetic crosslinking reagents, natural derived ones, such as genipin extracted from the *Gardenia jasminoides* fruit [92] have gather world-wide attention in the field of tissue engineering, mainly due to their low toxic potential and wide variety of active groups, such as ester bonds and hydroxyl groups that can react directly with the amino acid residues or proteins, maintaining the basic structure of the collagen support while also improving their biological stability [8]. Thus, genipin reacts non-specifically with primary amino groups, forming a secondary activated genipin whose ester groups then interact with proteins by creating secondary amide bonds [61]. Recent studies demonstrated that genipin-cross-linked collagen-based hydrogels maintain their structural integrity while supporting cell viability. For instance, Li et al. [93] developed a nanocomposite injectable collagen/chitosan hydrogel, with genipin as the crosslinking agent, for the delayed diabetic wound treatment. Different weight ratios of collagen–chitosan hydrogels were prepared, i.e., 50:50 (Gel 1), 25:75 (Gel 2), 0:100 (Gel 3). Notably, the collagen: chitosan hydrogel with a mass ratio of 25:75 (Gel 2) exhibited superior mechanical properties, swelling behaviour, and degradation rates. Thus, when compared to the other two tested formulations, Gel 2 displayed a swelling rate in PBS or approximately 1500% similar to Gel 3, but higher than Gel 1 (750%) and a degradation rate of 24% after 7 days in PBS (Gel 1—35%; Gel 3—11%). It is important to mention that after 2 weeks, Gel 2 was still present in a proportion of 15–20%, an observation which highlights the potential of this hydrogel as an ideal drug delivery platform. Moreover, the in vitro experiments indicated that Gel 2 is capable of sustaining cell viability and stimulating cell migration at the wound site. The animal studies further cemented the Gel 2 potential as an ideal wound dressing through its ability to promote collagen deposition, new blood vessel formation, and inhibition of MMP-9 expression. Similarly, Wu et al. [94] demonstrated the feasibility of a genipin cross-linked human recombinant type I collagen (rhCol I)/carboxymethyl chitosan (CMC) hydrogel loaded with exosomes derived from human umbilical cord mesenchymal stem cells (huCMSCs) as a potential therapeutic approach in severe skin wounds. The morphological and structural analysis of the newly developed hydrogels demonstrated a well organised porous structure (pore size in the rage of 100–200 μm), favourable for drug loading and its subsequent controlled release; an improved hydrophilicity, after CMC blending (*p* < 0.0001 vs. rhCol I hydrogel) and exosomes loading (*p* < 0.01 vs. rhCol I/CMC hydrogel); a superior swelling ratio in PBS and a stable degradation rate in both PBS and collagen of the rhCol I/CMC hydrogel. In terms of cytotoxic effects, the composite exosomes loaded hydrogel was capable of sustaining cell viability and promoting cell growth. Furthermore, the effects of the genipin cross-linked composite hydrogel on the full-thickness skin defect mice model showed that, compared to the control group, the rhCol I/CMC-Exos treated group presented a superior wound healing efficiency through an increase in fibroblast proliferation, modulation of inflammation, and angiogenesis promotion. In another study, Shagadarova et al. [95] developed genipin cross-linked collagen/chitosan hydrogels blended with silver nanoparticles (Ag NPs) coated with a chitosan derivative for wound healing in diabetic patients. The reported results revealed that the hydrogels comprising high molecular weight (700 kDa) chitosan and collagen (Ch700:Col 1:1-G) exhibited superior swelling properties compared to those formulated with low molecular weight chitosan (100 kDa) and collagen. Notably, after crosslinking, all hydrogel formulations displayed a fibrous, streak-like architecture with large pores and exhibited a 2-fold increase in storage modulus (G’), indicating a significant enhancement in their elastic properties. The inclusion of AgNPs further improved the mechanical performance, with both storage and loss moduli increasing by approximately 1.5 times in the Ch700:Col 1:1-G sample. In vivo wound healing experiments revealed that treatments using genipin-cross-linked hydrogels (with or without AgNPs) led to enhanced collagen deposition, improved regeneration of hair follicles, and reformation of sebaceous glands. Additionally, gene expression analysis indicated upregulation of VEGF, TGF-β1, IL-1β, and tissue inhibitor of matrix metalloproteinase (TIMP)-1, which are key mediators of tissue repair.

### 4.3. The Enzymatic Crosslinking Process

In contrast to the physical and chemical crosslinking processes, the enzymatic induced crosslinking method has attracted an intense interest in recent years due to its excellent specificity, mild reaction conditions, absence of secondary products, high yield, and enhanced catalytic efficiency [8,78]. Based on the type of catalytic reaction, the enzymatic cross-linkers can be categorized into transferases (glutamine transaminase), hydrolases (lysyl oxidase), and oxidoreductases (horseradish peroxidase) [65], which can modify the amino groups and produce protofibril bonds [96]. In a physiological environment, collagen undergoes several enzymatic post-translational modifications that are essential for its proper function and structural integrity, enabling the protein to maintain its structural integrity, elasticity, and biological activity [8]. One of the enzymes responsible for these modifications is glutamine transaminase, a transferase that catalyses the acyl transfer reaction between a γ-carboxyamide group of glutamine residue in protein and a primary amine, leading to the formation of inter- or intramolecular ε-(γ-glutamyl) lysine bonds and the covalent crosslinking of collagen. Another enzyme used as a collagen crosslinker is lysyl oxidase, a hydrolase involved in the modification of the ε-amino groups of lysine and hydroxylysine into aldehyde groups. The resulting aldehyde groups interact with the adjacent unmodified ε-amino groups, leading to the formation of a cross-linked collagen [65]. Lastly, horseradish peroxidase is a plant-derived enzyme capable of catalyzing the phenol-rich polymers via H_2_O_2_ consumption as an oxidant [97]. Since collagen is rich in tyrosine residues, the horseradish peroxidase-H_2_O_2_ system can oxidise them in order to generate active free radicals and crosslink the collagen fibres [98].

However, despite the lack of drawbacks that the physical and chemical crosslinking methods seem to suffer, the enzymatic crosslinking strategy is the most expensive technique, which is why its use is heavily limited.

### 4.4. Emerging Cross-Linkers and Green Chemistry Approaches

In recent years, concerns regarding the safety and environmental impact of traditional chemical crosslinking agents led to the development of various alternative cross-linkers that are both biocompatible and sustainable [99]. As mentioned before, conventional chemical cross-linkers such as glutaraldehyde, while efficient in improving the mechanical properties of the hydrogels, are limited by their cytotoxic effects and high environmental footprint [99]. As a consequence, research has shifted towards naturally derived, non-toxic alternatives that adhere to the green chemistry principles without compromising the therapeutic aspects. One example is citric acid, a water-soluble, naturally derived crosslinking agent, with low toxicity, good biodegradability, and low costs. Based on reported results from other types of hydrogels designed for skin wound treatment, the use of citric acid results in an improved hydrophilicity and structural stability of hydrogels. Camaci et al. [100] manufactured carboxymethylcellulose-poly(vinyl alcohol) hydrogels as innovative wound dressings and observed that by incorporating citric acid at 20% by weight of the polymer, the swelling ratio and water retention of the hydrogel increased up to 15 times their dry weight. Furthermore, emission scanning electron microscopy revealed that the developed hydrogels displayed an average porosity of 42.529% and pore sizes ranging from 3.429 to 32.620 μm, which are considered suitable for cellular growth and tissue regeneration. Given these outcomes, it can be reasonably expected that citric acid crosslinking would similarly enhance the mechanical integrity and porosity of collagen-based hydrogels, thereby improving their performance as wound healing materials. However, it is important to mention that the crosslinking with citric acid typically requires thermal activation at temperatures exceeding 80 °C, which may compromise the native structure of collagen and implicitly its biological functionality [101].

Tannic acid, a naturally occurring compound, has emerged as a multifunctional crosslinker offering a unique combination of mechanical reinforcement and therapeutic activity. As a polyphenolic compound, tannic acid forms non-covalent interactions within the three-dimensional collagen framework, thereby allowing the creation of supramolecular materials with an enhanced functional performance. Michalska-Sionkowska et al. [102] explored the potential of tannic acid as a crosslinking agent in the manufacturing of collagen/β-glucan hydrogels as potential wound dressings, and the overall results demonstrated the feasibility of tannic acid as a powerful, multifunctional crosslinker for collagen-based hydrogels. Thus, by varying the tannic acid concentration (2%, 5% and 10%), it was observed that by incorporating tannic acid in a 10% concentration, structurally stable hydrogels with better mechanical properties could be obtained. Additionally, the prolonged release of tannic acid was beneficial for the biological functionality of the hydrogel, leading to an efficient antimicrobial effect and an enhanced pro-regenerative activity. These findings highlight the importance of using the right concentration of crosslinking agent that could lead to a perfect balance between the mechanical performance and the therapeutic functionality of the resulting hydrogel, ensuring its suitability as an efficient wound dressing. Is it worth mentioning that the use of tannic acid is limited due to its non-specific binding to hydrogen binding acceptors, a phenomenon which leads to an amorphous structure with weak mechanical characteristics, as opposed to an ordered, natural structure [103]. Other promising candidates for wound healing applications are the epoxy-functionalized polymers, which offer antioxidant or redox-sensitive features beneficial in oxidative chronic wound environments. For instance, gelatin-based hydrogels cross-linked using dioxirane derivatives of polyethylene glycol, specifically poly(ethylene glycol) diglycidyl ether (PEGDE-500), show considerable promise as wound dressings. Thus, Maikovych et al. [104] designed and developed hydrogel materials using gelatin and PEGDE 500 as potential candidates for the treatment of chronic and infected wounds. By optimizing the gelatin–PEGDE-500 ratio between 1:3 and 1:5, highly elastic networks capable of preserving their structural integrity (even at 40 °C) were obtained. More than that, the study reported that the network’s pore dimension can be influenced by the crosslinker concentration, i.e., the 1:5 ratio led to smaller pores, modification also reflected into the low swelling ratios across physiologically relevant fluids (11.0 ± 1.8%—water, 8.8 ± 0.9%—saline, 8.7 ± 0.8%—exudate), while a lower gelatin–PEGDE-500 ratio (1:3) increased the pore dimensions (12 ± 5) and implicitly the swelling capacity (13.7 ± 2.1%—water, 10.5 ± 1.6%—saline, 9.1 ± 1.2%—exudate). In addition, these hydrogels demonstrated strong mechanical resilience, robust gel fractions, and complete cytocompatibility in vitro. When loaded with chlorhexidine, they released approximately 50% of the antiseptic within the first 24 h and effectively inhibited *Escherichia coli* and *Streptococcus aureus* growth. Overall, PEGDE crosslinking can represent a purification-free synthesis route for hydrogels that are not only affordable but also absorbent, cytocompatible, and antimicrobial. Although many epoxy crosslinkers are synthetic, recent developments in bio-based epoxy chemistries suggest increasing alignment with green chemistry objectives. Their use remains limited in collagen systems, but their potential for long-term wound coverage and payload delivery is noteworthy [101], and it could provide a new pathway for designing bioactive and eco-compatible collagen-based hydrogel systems. In a recent study by Tang et al. [105], the use of an epoxy-modified *Bletilla striata* polysaccharide (BSP) was explored as a crosslinking agent for collagen-based sponges, manufactured with the purpose of wound care. By incorporating the epoxy groups, a robust chemical crosslinking within the collagen matrix was observed, a phenomenon which led to the preservation of the triple-helix structure and the formation of a porous network sponge (85%). Additionally, the swelling ratio reached 1100% in PBS, highlighting the hydrogel’s high capacity for fluid absorption, which is crucial for maintaining a moist wound environment. More than that, the mechanical testing demonstrated the hydrogel’s suitability for tissue regeneration, revealing a compressive modulus of 9697 ± 24 Pa, indicative of its integrity and elasticity. The optimized formulation (0.5 mg/mL oxidized BSP with 5 mg/mL collagen) achieved a fast hemostasis, which required only 25 ± 4 s to stop the bleeding in rat models with liver injuries and reduced the blood loss to only 23.5 ± 4.95 mg compared to 126.7 ± 19.1 mg for gauze. As a whole, the reported results suggested that these epoxide-cross-linked collagen–BSP sponges exhibit strong mechanical properties, a hemostatic efficiency, antimicrobial activity, and biocompatibility, demonstrating their potential as next-generation wound dressings.

## 5. Properties of the Collagen-Based Hydrogels

### 5.1. Hydrophobicity and Moisturization

The hydrophilic nature and high water retention capacity of collagen-based hydrogels are two notable characteristics that result from the high proportion of hydrophilic functional groups found on the surface of the collagen molecule, making them not only hydrophilic and moisturising but also water-absorbent [106]. This exceptional hydrophilicity, coupled with their excellent biocompatibility, makes collagen-based hydrogels ideal candidates for a variety of clinical uses, i.e., skin wound dressings. Furthermore, depending on the intended biomedical application, the absorptive capacity of the hydrogel can be tailored to suit the desired practical use, leading to improvements in its performance and stability [106].

### 5.2. Mechanical Properties

As previously mentioned, the mechanical characteristics of collagen-based hydrogels are highly sensitive to an array of factors, including the collagen’s origin, type, and concentration, the used crosslinking technique, and the incorporation of additional materials (e.g., other polymers) [107]. For example, higher concentrations of collagen typically result in a much denser and more stable 3D framework, while the employment of the chemical crosslinking method can further improve the structural integrity of the hydrogel [106]. Furthermore, the mechanical performance of the hydrogel can also be further optimised through the inclusion of other polymers or nanofillers, which contribute to the formation of a more robust multi-network structure [107]. Therefore, by employing these strategies, not only are the durability and integrity of hydrogels significantly improved, but their mechanical properties can also be tailored to meet the specific demands of the application.

### 5.3. Degradability

The biodegradable nature of the collagen-based hydrogels represents one of their most important feature, which makes them highly attractive in the field of regenerative medicine. As a natural protein found in the human body, collagen is degraded by specific enzymes known as collagenases. These enzymes cleave the collagen fibres at specific peptide bonds, leading to smaller peptides or amino acids [108]. The resulting smaller peptides can be safely taken up and recycled by the circulatory system or be removed from the body via metabolic pathways [106]. In tissues like skin or cartilage, collagenases are naturally present, ensuring that the collagen-based hydrogel will degrade over time in a biologically controlled manner [107]. The degradation process is heavily impacted by a series of factors that include the collagen’s type, the used crosslinking agents, and the surrounding environment [109,110].

The degradation process of collagen-based hydrogels is a vital feature that dictates their use in clinical applications, as it directly impacts their effectiveness as (i) drug delivery platforms and (ii) optimal scaffolds for wound repair and tissue regeneration [109]. For example, in wound healing and tissue engineering, the degradation rate of collagen hydrogels should align with the rate of new tissue formation. According to Jiao et al. [111], a degradation half-life of approximately 14 days is considered optimal for cellular growth and skin tissue regeneration. Thus, if the degradation process happens too fast, the scaffold may not provide enough support for the growing tissue, while if the degradation rate is too slow, it could affect the tissue remodelling process [112]. Moreover, the collagen-based hydrogels have also demonstrated their remarkable potential as drug delivery systems, especially in applications where the timed and targeted release of the active substance is required [109]. Thus, a controlled degradation rate can be used to release drugs over time.

### 5.4. Swelling Properties

The swelling rate of collagen hydrogel relates to its volume expansion upon water absorption and is impacted by a wide range of parameters, including the environmental pH and temperature, the collagen’s concentration, and crosslinking method [113]. Amongst them, the cross-linking method is a key parameter that needs to be taken into account, as it affects the density of the polymeric network. Data reported in the literature demonstrated a direct relationship between the crosslinking degree and the swelling capacity of the hydrogel. Thus, an extended crosslinking results in a more compact network structure, which in turn leads to a low swelling rate due to a restricted polymeric chain mobility [114]. In order to be used with success in clinical applications, particularly in the treatment of chronic skin wounds, hydrogels should be able to achieve a swelling ratio in the range of 500–1500% of their dry weight, depending on the severity of the trauma and fluid volume. However, this property is only favourable to a certain extent, i.e., hydrogels that swell excessively (>2000%) may become unstable or cause maceration of the surrounding tissue, while hydrogels with a swelling degree under 300% are not capable of providing a moist environment favourable for an optimal wound healing [115]. It is important to mention that data reported in the literature may not always reflect the in vivo conditions and can lead to conflicting results due to the environment in which the measurement was performed. For example, deionised water differs from biologically relevant media such as PBS, Ringer’s solution, and synthetic wound exudate due to the presence of ions, proteins, and pH variability. Literature reports that collagen-based hydrogels cross-linked with genipin or tannic acid exhibit swelling ratios of 800–1200% in PBS but significantly lower in Ringer’s solution, up to 400–600%, mainly due to the ionic interactions, which may affect the water uptake [116,117,118,119]. Therefore, the degree of swelling should be evaluated under physiologically relevant conditions and tailored for the intended wound type. Additionally, the swelling capacity is a direct indicator of the polymer network hydrophilicity and crosslinking density, and it can be used as a criterion for batch-to-batch variations and consistency in hydrogel fabrication [120]. Consequently, by exercising precise control over these factors, the hydrogel’s degree of swelling can be tightly regulated.

### 5.5. Self-Healing

**Figure 6:**
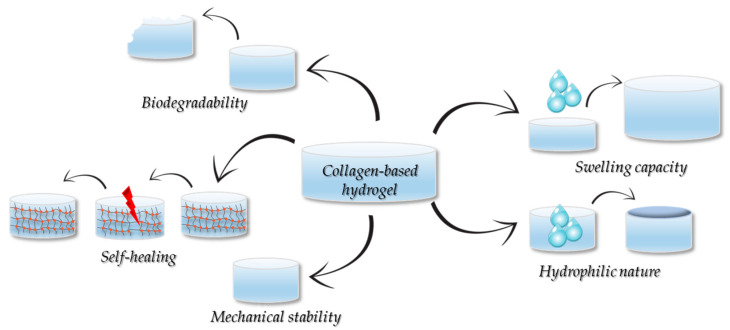
The schematic illustration of the properties of collagen-based hydrogels designed for application in skin wound healing.

Due to the high number of reversible and dynamic chemical bonds (i.e., imine, borate, hydrazine, disulfide) present in their network structure, the collagen-based hydrogels exhibit a self-repair ability, which enables the hydrogel to recover its original structure and function after damage, via the breaking and re-formation of the chemical bonds [121,122]. Gu et al. [123] prepared a collagen-chitosan hydrogel cross-linked with oxidation-modified konjac glucomannan (OKGM) in which AgNPs were dispersed, and the principle of Schiff-based reactions was utilized to create a self-healing, typical injectable hydrogel. Thus, dynamic Schiff base bonds formed between the aldehyde groups of OKGM and the amino groups of both collagen and chitosan create a reversible and flexible hydrogel network. The in vitro and in vivo results demonstrated the potential of the newly developed construct as a dressing material for larger wounds with irregular shapes. Figure 6 denotes the properties of the collagen-based hydrogels dedicated to skin wound healing.

## 6. Collagen-Based Hydrogels for Skin Wound Healing

Due to their high water content of up to 70–80%, collagen-based hydrogels are exceptionally well suited for the management/treatment of various skin wounds. Their semi-permeable nature permits the controlled exchange of both gases and liquids, which enables the autolytic debridement of the existing necrotic tissue by creating a moist wound environment, important for the enzymatic activity involved in the autolysis process [7]. Moreover, their soft and adjustable texture allows easy trimming for an exact fit to the wound site, and their natural transparency simplifies the healthcare providers’ work by allowing them to observe the condition of the wound without having to constantly remove the dressing [124]. Furthermore, their non-adherent performance ensures that upon removal, no secondary injury occurs, and no residue is left behind, while their soothing cooling effect can significantly lower the post-surgical pain and inflammation [125].

**Figure 7:**
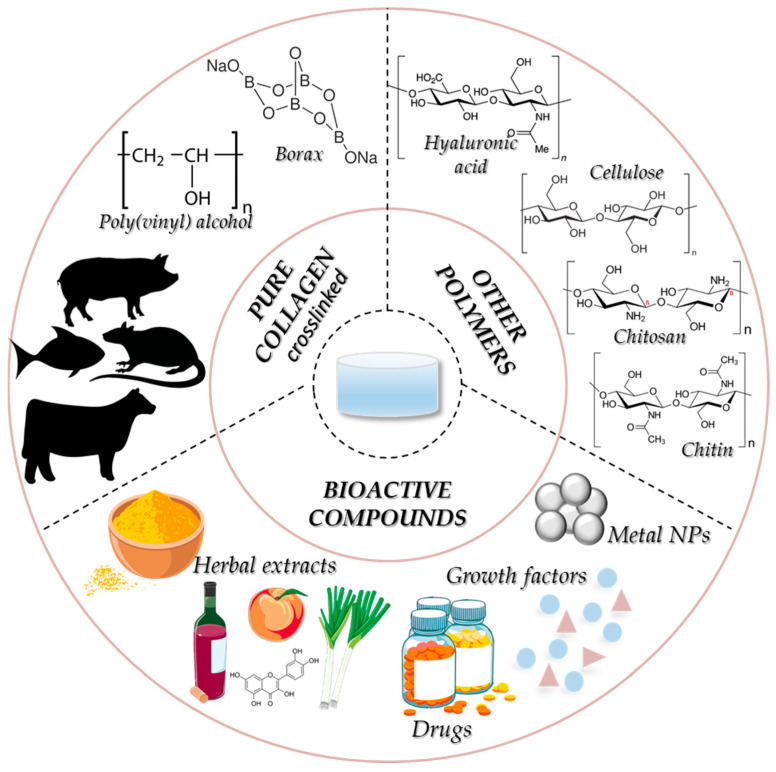
The schematic illustration of the three main categories of collagen-based hydrogels designed for skin wound applications: pure collagen; collagen blended with bioactive molecules (i.e., herbal extracts, metal NPs, drugs, cell-penetrating peptide, growth factors, cytokines, etc.); and collagen blended with other natural and/or synthetic polymers (i.e., chitosan, hyaluronic acid, cellulose, etc.).

In line with recent research, the collagen-based hydrogels for skin wounds can be categorised as (Figure 7) (i) pure collagen; (ii) collagen blends with bioactive molecules (i.e., herbal extracts, metal NPs, drugs, cell-penetrating peptide, growth factors, cytokines, etc.) for drug delivery; (iii) collagen blends with other natural and/or synthetic biopolymers (i.e., chitosan, hyaluronic acid, cellulose, etc.) [8,23].

The following section will review recent research into collagen-based hydrogels as a therapeutic strategy for chronic wounds, such as diabetic foot ulcers (DFUs), pressure ulcers (PrUs), and venous leg ulcers (VLUs). Diabetic foot ulcerations represent a prevalent complication in elderly diabetic individuals, and are characterised by epidermal lesions, ECM destruction, and ultimately loss of tissue integrity and amputation of the affected limb [15]. Similarly, VLUs are chronic injuries with a high prevalence among older people, characterized by open wounds in areas affected by hypertension, such as the lower tibia [126]. Individuals with VLU exhibit symptoms such as pain, heaviness of the affected limbs, varicose veins, gravitational dermatitis, subcutaneous fibrosis, cutaneous hyperpigmentation, and dermal weeping [127]. With high prevalence in individuals with a reduced activity and mobility, pressure ulcers are defined as localised skin or subcutaneous tissue lesions that occur in the area of bone protuberance (i.e., heels, foot, hips, ankles, shoulders, elbow, coccyx, ear flaps) caused by continuous pressure coupled with shear and/or frictional forces [128]. Individuals with severe PrUs are susceptible to secondary infections that can lead to sepsis, skin cancer, and ultimately death [15].

In light of the multifaceted nature of chronic wounds, collagen-based hydrogels emerge as a promising avenue for innovative therapeutic intervention.

### 6.1. Simple Collagen-Based Hydrogels for Chronic Skin Wounds

As mentioned before, collagen-based hydrogels can be blended with various bioactive molecules or polymers in order to improve their biological performance, or in some instances, they can also be used in their pure form, without the addition of any bioactive agents or polymers [8]. Data reported in literature revealed that collagen in its purest form is safe and that simple collagen-based hydrogels can be used as efficient dressings for varying cutaneous wounds. For instance, in a study by Ge et al. [129], acid-soluble collagen (ASC) and pepsin-soluble collagen (PSC) were extracted from Tilapia skin and characterised with the purpose of choosing the most attractive material for hydrogel manufacturing. By using sodium dodecyl sulphate-polyacrylamide gel electrophoresis (SDS-PAGE), differential scanning calorimetry (DSC), circular dichroism (CD) and Fourier transform infrared spectroscopy (FTIR) analysis, significant differences between the two collagens were observed, i.e., PSC displayed a lower molecular weight and thermal stability (PSC—50.57 °C; ASC—51.59 °C), an isoelectric point closer to neutral (PSC—5.75; ASC—5.40), and an inherent low antigenicity. These findings suggested that PSC can self-aggregate faster to form hydrogels within a neutral pH range and is more easily degraded and absorbed in an in vivo environment. Based on this, four types of PSC-based hydrogels were fabricated, with different PSC concentrations (5 mg/mL, 10 mg/mL, 15 mg/mL, and 20 mg/mL). The physicochemical analysis revealed that the hydrogels’ properties were concentration-dependent, with the water retention ratio increasing with PSC concentration. This effect was attributed to the expansion of the hydrogels’ mesh structure with higher PSC content, resulting in denser collagen networks capable of trapping water molecules more effectively. As a result, the retained water could not be easily released without the application of centrifugal force. The rheological analysis confirmed the water retention observations, suggesting that the 10 mg/mL and 20 mg/mL PSC-based hydrogels displayed the best mechanical properties due to an increase in the microstructure of the collagen fibres. Furthermore, the in vitro examination revealed a good biocompatibility and no cytotoxic effects on the NIH-3T3 cells, while the animal studies on deep second-degree burns in rat models showed that after 14, 21 and 28 days, the healing process in the 10 mg/mL PSC-based hydrogel group was significantly improved in comparison to both the blank control (no treatment after wound) and the used commercially available product (3MTM Tegaderm^TM^). Similarly, Li et al. [130] designed and fabricated a self-healing injectable codfish-derived collagen-peptide-based hydrogel, comprised of collagen-adipic acid dihydrazide functionalised collagen peptide (Col-ADH), oxidized dextran (ODex), and different ratios of polyvinyl alcohol (PVA) and borax, denoted as PBCO-1, PBCO-2, PBCO-3, PBCO-4, as potential dressings for skin wound healing. Compared to the PCBO-1 hydrogel (lacking PVA and borax) where the SEM images revealed a slightly brittle skeleton with large pores, the introduction of borate ester bonds in the hydrogels’ structure resulted in denser, more elastic skeletons, with pore sizes and porosities dependent on the mass ratio of PVA–borax (PBCO-1 (84.7%) > PBCO-4 (82.0%) > PBCO-3 (79.1%) > PBCO-2 (75.2%)). In terms of water retention ratio, the PBCO-1 hydrogel (lacking PVA and borax) exhibited the lowest water retention (48.1%), while the PBCO-2 hydrogel (8:1 PVA–borax) presented the highest value (85.4%). This can be attributed to the increased crosslinking density resulting from the increase in the borate ester bonds and hydrogen bonding interactions, which led to the formation of a more compact and interconnected hydrogel network. More than that, the presence of the multiple-dynamic-bond cross-linked network endowed the PCBO-2 hydrogel with good mechanical (12 kPa at 60% strain) and adhesive (5.28 kPa vs. Greenplast^®^-5 kPa; on porcine skin) strength, and an excellent self-healing ability and injectability. In terms of antibacterial effects, the introduction of negatively charged borate bonds, along with collagen peptides, resulted in an effective antibacterial activity against both Gram-negative and Gram-positive bacteria. The results of cytotoxicity, hemolytic activity, hemostatic ability, cell migration, and in vivo wound repair demonstrated the suitability of PBCO-2 hydrogel as an ideal dressing for refractory wounds. Taken together, these results highlight the importance of borate ester bonds and hydrogen bonding interactions introduced by PVA and borax and their vital role in the mechanical properties and biological functionality of the resulting hydrogels. Another dual-dynamic-bond cross-linked fish-derived collagen-based hydrogel for skin wound healing was also developed by Cai et al. [131]. Thus, hybrid hydrogels comprised of fish-skin collagen, borax, PVA, and different concentrations of oxidized sodium alginate (OSA), namely 10 mg/mL, 20 mg/mL, 40 mg/mL (denoted COSP10, COSP20, and COSP40), were developed. SEM analysis showed a hydrogel network structure dependent on OSA content, with higher OSA concentrations leading to a more compact hydrogel structure with a reduced pore size. These observations are a clear indication of the fact that the dual-dynamic-networks are responsible for an increase in the number of intermolecular crosslinking points and, implicitly, of the crosslinking density of the hydrogel. In addition, the synergistic effect of OSA hydrophilic nature and PVA’s efficient interaction with hydrogen bonds in the water molecules resulted in an increase in water content for the hydrogels with high concentrations of OSA (COSP40—25.37± 0.79%; COSP20—20.39 ± 0.92%; COSP10—18.62 ± 0.41%), as measured after 21 days in PBS. Based on these results, the COSP20 hydrogel was considered the optimal solution for in vivo applications, which is why the in vivo examination was carried out using only the COSP20 hydrogel. Therefore, in an vivo environment, the COSP20 hydrogel was capable of promoting wound closure in a faster rate than the control groups, i.e., on 14th day of treatment, the healing rate of the COSP20 hydrogel group was 95.54 ± 3.92% of the gauze group was 73.33 ± 3.30%, while that of the commercial dressing group was 89.36 ± 2.70%. Through the introduction of dual-dynamic-bonds and intramolecular hydrogels interactions between the fish collagen, OSA, borax and PVA, the limitations of marine-derived collagens in terms of denaturation, mechanical strength, stiffness and bioactivity haven been overcome, thus leading to an increased potential of fish-skin collagen as suitable candidates in the production of hydrogels as skin wound dressings. In another attempt to improve the properties of collagen-based hydrogels using OSA, a source of dynamic covalent bonds, multifunctional hydrogels composed of varying concentrations of collagen and OSA were fabricated (OSACol(15), OSA-Col(20), OSA-Col(25), and OSA-Col(30)) using a biphasic solvent system of acetic acid/1-ethyl-3-methylimidazolim acetate (AA/[EMIM][Ac]) [132]. Through the use of this highly polar solvent system, the electrostatic interactions between the oppositely charged polyelectrolytes are minimised, thus facilitating the fabrication of a compatible hydrogel network. The resulting hydrogels exhibited desirable physico-chemical properties, such as a robust mechanical strength (~1008 Pa), enhanced thermal stability (~66.6 °C), reduced degradation (~23.5%), and a well-defined porous microstructure (~44 μm). In addition to their structural advantages, the developed hydrogels showed excellent injectability, self-healing behaviour, excellent biocompatibility, and were capable of significantly improved wound healing efficacy. In another take at tailoring the properties of collagen-based hydrogels to suit the requirements essential for an optimal wound healing process, Aguayo-Morales et al. [133] developed innovative semi-permeable collagen-polyurethane-dextran-based hydrogels, in which the dextran was incorporated at different concentrations (10 wt.% (D10), 20 wt.% (D20), 30 wt.% (D30), equivalent to 0.6 mg, 1.2 mg, and 1.8 mg, respectively). Therefore, the introduction of dextran into the formulations led to an improvement in the crosslinking index of the collagen- polyurethane (PU) hydrogels (measured at 35%) and, implicitly, in their swelling capacity (2800–3000% in PBS), mainly due to the numerous glyosidic-OH groups within the branched structure of the polysaccharide. In terms of mechanical properties, the oscillatory rheology analysis revealed that the hydrogel containing 20 wt.% dextran (D20) presented more robust interactions formed by hydrogen bonds created between the amide and hydroxyl groups within the collagen-PU matrix and the glyosidic–OH bonds of dextran, which resulted in an increase in its storage modulus (8900 Pa vs. 3200 Pa—D30 and 6900 Pa—D10). In addition, D20 hydrogel displayed the best biological activity, being able to sustain cell viability and promote fibroblast proliferation, while tuning the inflammatory activity towards an anti-inflammatory phenotype in macrophages. It is important to mention that the in vivo results suggested that the D20 hydrogel was capable of accelerating wound healing in diabetic rats by stimulating collagen fibre deposition and promoting new blood vessel formation. Thus, the obtained formulations can represent plausible candidates as long as the optimization of the dextran concentration enclosed in the hydrogel matrix is finely controlled so that the interaction between the polysaccharide and the crosslinking agent will lead to improvements in the hydrogel’s properties.

### 6.2. Collagen-Based Hydrogels Loaded with Bioactive Molecules for Chronic Skin Wounds

A step ahead of simple collagen-based hydrogels involves the incorporation of various bioactive molecules into the collagen matrices to enhance their therapeutic efficacy. This approach not only boosts the mechanical properties of the hydrogels but also imparts a range of beneficial biological activities. Metal NPs, plant extracts, pharmaceutical agents, and growth factors are perfect examples of bioactive molecules that, once blended into the collagen-based hydrogels, can significantly contribute to the wound healing process by mitigating the risk of microbial infection, reducing the pro-inflammatory responses, and promoting tissue regeneration. The following section will explore studies that focus on the integration of these bioactive agents into collagen-based hydrogel systems.

Metal NPs are three-dimensional crystalline materials with a high surface area-to-volume ratio that can be synthesised in a wide variety of shapes and sizes, each exhibiting different physicochemical and biological activities [134]. Based on their strong stability, high loading capacity, specific targeting, and trigger release abilities, metal NPs have been used extensively as both drug delivery vehicles and carriers [134]. In wound healing, the use of these metal NPs is based on their ability to trigger neutrophil apoptosis, reduce the mitochondrial membrane potential, and consequently reduce the cytokine production [135]. In addition, metal NPs promote wound repair by promoting the proliferation and migration of epidermal cells and fibroblasts, primarily through photothermal effects [136]. Furthermore, they inhibit the growth of various microorganisms, exhibiting a superior antibacterial activity, a feature which makes them particularly valuable in preventing wound infections [137]. In this context, collagen-based hydrogels blended with metal NPs represent an outstanding progress in the field of wound management and skin regeneration. In comparison to pure collagen matrices, these innovative blended hydrogels present improved physical and mechanical characteristics due to the distribution of the NPs, and exhibit an array of biological activities, e.g., a broad-spectrum antibacterial effect, low immunogenicity, and pro-regenerative abilities [8,134]. Hu et al. [138] developed a recombinant human collagen type III (rhCol III)-based hydrogel blended with Ag-loaded polydopamine NPs (PDA@AgNPs) with the purpose of a controlled release of the NPs, and the reported results showed that the hydrogel provided a strong antibacterial effect and was capable of reducing the inflammatory response due to the gradual release of the AgNPs. Furthermore, the subsequent release of these NPs promoted cell migration and proliferation. Moreover, the in vivo study showed that after 14 days, the hydrogel group achieved a 98% wound healing rate in type II diabetic rat models. In another study, Zhang et al. [139] prepared a collagen/chitosan-based hydrogel incorporated with AgNPs obtained through the bioreduction of Ag^+^ and compared its biocompatibility and antibacterial activity with a hydrogel incorporating Ag^+^ and commercially available AgNPs. The results demonstrated the superiority of the newly developed COCAgNP hydrogel. In addition, animal studies showed that the hydrogel significantly speeds up the healing process of the infected full-thickness skin wounds by stimulating collagen formation, suppressing the inflammatory response, and supporting the processes of re-epithelialization and new blood vessel growth. Fu et al. [140] developed an injectable antibacterial hydrogel composed of type I collagen (Col I) and AgNPs at two concentrations—0.588 mM (Col I/AgNPs-1) and 2.49 mM (Col I/AgNPs-2)—as a potential treatment for full-thickness diabetic wounds. Physicochemical analysis showed that incorporating AgNPs significantly enhanced the hydrogel’s mechanical and structural properties. Specifically, the storage modulus increased from 36 Pa (Col I) to 107 Pa and 272 Pa for the AgNP-loaded variants, indicating improved elasticity. Additionally, the modified hydrogels exhibited larger pore sizes, faster water absorption (reaching equilibrium in 3 min vs. 30 min for pure Col I), and a slower degradation rate (<77% for AgNPs-loaded vs. 91% for pure Col I), all of which support their suitability for wound healing applications. Notably, the in vitro observations highlighted the antibacterial and pro-regenerative effects of the hydrogels, while the in vivo study demonstrated their ability to accelerate diabetic wound healing, with Col I/AgNPs-2 (2.49 mM AgNPs) showing the best outcomes. By day 14, Col I/AgNPs-2 achieved nearly complete wound closure with no visible wound gap, compared to 3.06 ± 0.07 mm in the control group (untreated wounds) and 2.65 ± 0.09 mm in the Col I group. Wound closure rates exceeded 55% on day 4 and 80% on day 7 for both AgNP-loaded groups, significantly outperforming controls (<30% on day 4, <60% on day 7). Histological analysis revealed fully developed connective tissue, complete re-epithelialization with skin appendages, reduced inflammation, and enhanced collagen deposition in the Col I/AgNPs-2 group, confirming its superior regenerative capacity. Alongside AgNPs, zinc oxide (ZnO) and copper oxide (CuO) NPs have also been incorporated into collagen-based hydrogels, as demonstrated in the study by Birca et al. [141]. In this work, three distinct formulations were developed: a control hydrogel (H_Mctrl) and two nanocomposite variants (H_MZnO and H_MCuO). FTIR analysis confirmed the preservation of the collagen’s molecular structure in all samples, while additional spectral bands in the 500–1000 cm^−1^ region verified the successful integration of ZnO and CuO NPs. SEM imaging further revealed a uniform distribution of multilayered microspheres embedded within a porous scaffold architecture, an essential characteristic for supporting tissue regeneration. Swelling tests indicated that H_MZnO exhibited the highest liquid absorption over 24 h, followed by H_MCuO and the control, a trend that was consistent with their degradation profiles, all remaining below 18%, with H_MZnO demonstrating the highest structural stability. Collectively, these results highlight the hydrogels’ robust structural integrity, functional adaptability, and significant potential for use in advanced wound healing applications. To highlight the potential of these materials for biomedical applications, their biological functionality was also assessed in terms of antimicrobial activity, cytocompatibility, and ability to promote tissue regeneration. Thus, ZnO- and CuO-loaded collagen hydrogels exhibited an effective antimicrobial activity, with H_MCuO outperforming against *Pseudomonas aeruginosa* due to higher ROS production and Cu^2+^ release. The biocompatibility evaluation revealed the mild cytotoxic effect of H_MCuO, indicating the need for further formulation optimization.

Apart from metal NPs, another example of bioactive agents that have been extensively incorporated into collagen-based matrices is natural compounds found in various herbal extracts. It is a well-known fact that a proper inflammatory response is required for an optimal healing process and that an excessive inflammatory activity can result in high levels of ROS, increased oxidative stress, and ultimately cellular death [142]. From data found in literature, it became apparent that a wide selection of herbal extracts exhibit antioxidant properties that can protect the skin tissue from oxidative damage [143]. Therefore, in order to maintain ROS levels at non-toxic concentrations and a proper redox balance, different bioactive compounds extracted from a wide array of medicinal plants have been employed with the purpose of endowing hydrogels with antioxidant properties that can encourage wound repair [142]. An example of a collagen-based hydrogel blended with an antioxidant compound is represented by the impregnated curcumin collagen-derived hydrogel developed by Shen et al. [144] for full-thickness burn wound healing. Curcumin is a natural bioactive compound extracted from the rhizome of the *Curcuma longa* plant, with antibacterial, anti-inflammatory, and antioxidant properties, used with prevalence in wound healing studies [145]. Due to its extremely low water solubility, hydrogel matrices represent suitable carriers for the sustainable delivery of the substance at therapeutic levels to enhance its bioavailability [142]. Therefore, through the use of this newly designed hydrogel, the restoration of the skin’s structure and function was significantly enhanced, with a wound closure period of only 9 days as opposed to the control group that was still in the re-epithelialization stage. Xiang et al. [146] investigated the effect of an amniotic membrane-derived collagen-based hydrogel loaded with quercetin on wound healing in diabetic rats and their findings indicated that, relative to the control group, the animals treated with the newly developed wound dressings presented an enhanced wound contraction rate, an increase in the epidermis and dermis volumes, TGF-1β and VEGF synthesis, new blood vessels, as well as an amplified collagen deposition. The study’s observations are not surprising at all, considering that quercetin, a flavonoid substance abundant in vegetables and fruits, exhibits an antioxidant activity, and that data reported in literature demonstrated its inhibitory role in both acute and chronic phases of inflammation [147,148]. Another study employed the use of *Continus coggygria* extract in the preparation of an atelocollagen-based hydrogel for chronic wound management, and the reported data revealed that this hydrogel was capable of generating a favourable microenvironment for optimal re-epithelialization and granulation tissue development during the wound healing process [149].

Furthermore, collagen can also be combined with hydrophobic drugs for targeted drug delivery. For example, in a study by Olivetti et al. [150] collagen hydrogels were grafted with dodecenylsuccinic anhydride (DDSA) to deliver simvastatin, a hydrophobic drug that exhibits anti-inflammatory properties. For this purpose, the varying concentrations of DDSA were grafted into collagen through two different methods: esterification after the gelation step and manufacturing of a hybrid gel during fibrillogenesis via ammonia vapour exposure. By employing SEM analysis, it was observed that the incorporation of DDSA during the gelation process impeded the self-assembly of collagen into fibrils, phenomenon attributed to the denaturation of collagen due to the reaction conditions (high temperature, ethanol presence). Since the preservation of collagen’s triple helix structure is crucial in the wound healing process, the hybrid materials were discarded, and the examination was carried only with the hydrogels in which DDSA was added after gelation (3% DDSA–collagen; 6% DDSA–collagen). In terms of hydrophilic behaviour and water retention ratio, the contact angle measurements revealed a higher water droplet angle in DDSA–collagen gels, consistent with the swelling assay, in which water absorption was 5.2 g/g for collagen and 1.9 g/g for the DDSA–collagen scaffolds, respectively. Additionally, the biological examination showed that the concentrations of pro-inflammatory cytokines decreased when simvastatin was incorporated, but the addition of DDSA lowered the cellular attachment and proliferation. However, despite the slight reduction, the developed construct exhibited a favourable biocompatibility and no-cytotoxic effects. Altogether, incorporating DDSA into the hydrogel formulation increased hydrophobicity, allowing greater loading of simvastatin. This, along with the observed slower release profiles, highlights their potential as effective delivery systems for poorly water-soluble drugs. Feng et al. [89] incorporated bacteriocin and polymyxin sulfate B into collagen-based hydrogels, and the mixed constructs were shown to exhibit an efficient antibacterial activity and promote a slight acceleration in the wound closure rate after 7 and 14 days of treatment. Moreover, Jia et al. [151] demonstrated the wound healing potential of hyaluronic acid (HA)/collagen-based hydrogels blended with metformin microspheres. The in vitro investigations, showed that the hydrogels suppressed the proliferation of macrophages, reduced the inflammatory activity via the macrophage switch in the polarization phenotype (from M1 to M2), stimulated the migration of fibroblasts and promoted the wound healing process. In addition, cytokines or growth factors can also be used to enhance the wound healing process. For instance, Zhang et al. [152] investigated the potential of an injectable collagen/polyethylene glycol-based hydrogel loaded with the stem cell factor (SCF) from the umbilical cord and the results indicated a pronounced new blood vessel formation, an increased collagen deposition and a macrophage transition towards the anti-inflammatory M2 phenotype which translates into a reduced inflammatory response. Thus, taken together the newly developed hydrogel can represent an ideal scaffold material with the potential to be used as a dressing for diabetic foot ulcers. In another study, Suliman et al. [153] incorporated microspheres loaded with stromal derived factor (SDF)-1α into a collagen-based hydrogel derived from the amniotic membrane. Overall, the findings demonstrated that by treating the diabetic wounds with the loaded collagen-based hydrogel, an accelerated wound healing process was achieved. This phenomenon was attributed to the hydrogel’s ability to establish a chemoattractant microenvironment that facilitates cell recruitment and their active participation in tissue regeneration at the wound site.

### 6.3. Composite Collagen/Polymer-Based Hydrogels for Chronic Skin Wounds

As mentioned in Section 5.1. the wound healing capacity of the collagen-based hydrogels can be drastically improved when other polymeric substances are added. For instance, the addition of chitosan into the collagen matrix can improve the therapeutic effect of the dressing by creating a hydrogel with enhanced mechanical properties and antibacterial, anti-inflammatory and pro-regenerative activities [154]. Moreover, compared to pure collagen, the hybrid hydrogel generates a more compatible microenvironment for cells, thus leading to an improved wound healing process [155]. Cao et al. [98] investigated the wound healing potential of double cross-linked human-like collagen (HCL)-carboxymethylated chitosan (CCS)-based hydrogel, and the results showed that, in comparison to the control gel comprised of gelatine, the novel collagen-chitosan hydrogel met the standards of biological materials by exhibiting excellent mechanical properties and an excellent biocompatibility. Thereby, the addition of gelatin into the collagen matrix slightly reduced the porosity of the hydrogel, from 90.1% in the HLC-CCS hydrogel to 85.7% in the gelatin-CCS variant, while enhancing from 1100% to 1325%. These changes indirectly reflect the internal structure, where higher swelling ratios suggest a greater surface area-to-volume ratio and faster equilibrium times point to a more interconnected network. The combination of HLC and CCS contributes to the improved mechanical properties of the hydrogel, primarily through the formation of stable chemical and hydrogen bonding interactions. Moreover, full-thickness skin injury experiments demonstrated that rats treated with the HCL-CCS hydrogel achieved complete restoration of skin structure and function in a shorter period compared to those in the control (untreated wounds) and gelatin hydrogel-treated group. In addition, the immunohistochemical staining demonstrated the ability of the HCL-CSS hydrogels to stimulate the expression of various immune mediators such as CD31 and VEGF, involved in the wound repair. Similar, Zhang et al. [156] synthesized a ternary hydrogel system based on PVA, chitosan and collagen with the purpose of increasing the antibacterial properties and bioactivity of the collagen hydrogel and the animal experiments suggested that the wound healing rate of the hydrogel reached 98.2% after 14 days due to its excellent mechanical properties, efficient antibacterial activity and optimal biocompatibility. In another study by Demeter et al. [157] multi-component collagen based-hydrogels composed of collagen, chitosan, and carboxymethylcellulose (CMC) as natural moieties, and poly(vinylpyrrolidone) PVP and polyethyleneglycole (PEG)/polyethylene oxide (PEO) as synthetic backbone, were designed and prepared by e-beam crosslinking. The newly developed hydrogels were investigated in terms of their physico-chemical, mechanical, structural morphological and biological properties and the reported results indicated that these hydrogels were stable, non-adherent when handled. Most importantly, they exhibited an elastic structure, with a storage modulus of 9.697 ± 24 Pa (PD81), which is ideal for skin wound applications. Furthermore, the homogeneous pore distribution indicates their potential as targeted drug delivery platforms for various bioactive molecules. In terms of biological properties, the in vitro testing showed that they were well tolerated by the healthy cells (VERO cell line), therefore further highlighting their promising potential as drug delivery systems. In addition, the nanocomposite hydrogels can also be loaded with one or more bioactive substances to promote rapid wound healing. One example is the collagen/chitosan hydrogel developed by Li et al. [93], which was blended with phycocyanin NPs loaded with a small molecule inhibitor of MMP-9–ND-36, for a rapid wound healing of chronic foot ulcers. The in vitro experiments demonstrated that the newly designed hydrogel was biocompatible and capable of stimulating in situ cell migration, while the animal experiments indicated that the group treated with the collagen/chitosan matrix exhibited an improved collagen deposition and angiogenesis by effectively inhibiting the expression of MMP-9. Ather hybrid collagen/chitosan dressing functionalized with propolis-ZnO NPs for a rapid wound healing was designed and fabricated by Zayed et al. [158]. The reported findings indicated that after treating the full-thickness injuries for 14 days, the ratio of the wound closure area increased to 93.31%. In addition, the histological examination revealed a newly formed tissue characterized by a rich network of newly formed blood vessels, absence of inflammatory cells, and a substantial increase in collagen deposition. Furthermore, the biochemical assessment of the endogenous antioxidant enzymes (superoxide dismutase (SOD)) and lipid peroxidation levels (malondialdehyde (MDA) content) highlighted an increase in SOD, which could trigger the wound healing process and a suppression of MDA, a possible cause of wound healing delay.

In order for the structure and functionality of the skin to be maintain, ECM relies heavily on the presence of a wide range of components such as type I collagen, HA, fibrin, etc. [159]; thus, by designing hybrid hydrogels that incorporate natural components of the ECM, the cutaneous wound healing process can be significantly improved and accelerated. Hyaluronic acid is a natural polysaccharide with excellent water-retaining capacity, attributed to its anionic structure and high affinity for cations. It can interact with a wide variety of cell-surface receptors, supporting cell adhesion, signalling, and the regulation of cellular behaviour [160]. Compared to pure collagen, matrices comprised of collagen and HA exhibit improved mechanical features (e.g., increased stiffness and postponed strain hardening) [161] and low immunogenicity. Liang et al. [162] reported that the newly developed composite RHAg hydrogel system, consisting of methacrylated recombinant human collagen (RHCMA) and methacrylated hyaluronic acid (HAMA), demonstrated key properties favourable for wound repair. The developed hydrogels exhibited a highly interconnected porous architecture, with porosity reaching approximately 60%, closely resembling native tissue and promoting nutrient exchange. The pore sizes ranged from 20 to 30 µm in single-component gels to a more optimal 11–15 µm in the RHCMA-HAMA composites, ideal for fibroblast infiltration and elongation. Swelling studies revealed excellent fluid absorption, with the RHAg hydrogels rapidly taking up exudate, an essential feature for maintaining a moist wound environment. Altogether, these features, combined with the integration of ultrasmall AgNPs (~4.35 nm), make the composite hydrogels promising candidates for advanced wound healing applications. In addition, the hydrogels exhibited an excellent bacteriostatic effect against *Streptococcus aureus* and *Pseudomonas aeruginosa*. Moreover, the in vitro study highlighted the high efficiency of the hydrogels on the adhesion, migration, and proliferation of the NIH3T3 cells. Additionally, in an in vivo microenvironment, the newly designed hydrogel was capable of regulating the inflammatory response, increasing collagen fibrillogenesis, and sustaining tissue regeneration. In another study, Bindi et al. [159] fabricated a biomimetic hydrogel based on collagen, HA and fibrin and the reported results showed that the formulated dressing exhibited viscoelastic properties (10 kPa) similar to those of native skin (1–10 kPa), an enhanced porous structure (average pore size of 120 µm) and an excellent biocompatibility when compared to the cellular monolayer grown onto tissue culture plastic (control). The in vitro observations demonstrate the importance of a biomimetic environment provided by the naturally derived biopolymers. In their study, Yang et al. [163] developed an injectable, self-healing hydrogel composed of collagen and HA with inherent antioxidant properties, which demonstrated its potential in effectively neutralizing excessive ROS concentrations, enhancing vascular cell proliferation, and improving the inflammatory microenvironment, thereby promoting an accelerated wound healing process. Due to the presence of boronic ester-based covalent bonds, the developed hybrid hydrogel presented a great flexibility, an excellent self-repairing ability and an increase adhesion (from 3.51 ± 0.31 kPa to 4.13 ± 0.22 kPa, after the content of PA was increased, which led to an enhancement in hydrogen bonding between hydrogel and the skin tissue). Moreover, the use of natural polymers endowed the hydrogel with superior biocompatibility, antioxidant properties, and the ability to promote cell proliferation and migration. Additionally, the animal experiments showed that the novel hydrogel accelerated wound repair through the promotion of the angiogenic process, collagen deposition, and the reduction of the inflammatory response.

In conclusion, the incorporation of complementary natural and synthetic polymers into collagen-based hydrogels noticeably amplifies their therapeutic potential for chronic skin wound healing applications. These modifications improve key hydrogel’s properties such as mechanical strength, porosity, swelling capacity, and biocompatibility, while also introducing beneficial biological functions like antibacterial, antioxidant, anti-inflammatory, and pro-regenerative activities. By optimizing the internal structure and enabling a controlled drug release, these hybrid hydrogels may generate a more favourable microenvironment for tissue repair, in which certain processes such as cell adhesion, proliferation, and angiogenesis are significantly enhanced. Taken together, these findings demonstrate that the strategic integration of additional polymers can transform collagen-based hydrogels into advanced, multifunctional platforms ideal for chronic wound care.

## 7. Conclusions and Future Directions

The ineffective management of acute wounds often leads to chronic wounds, which present a significant challenge in dermatology. The development of robust, durable, and efficient wound dressings represents an essential requirement for the optimal treatment of patients suffering from various chronic skin wounds such as DFUs, PrUs, or VLSs. In this context, various biomaterials have been employed in the design and development of wound-healing dressings, amongst them, collagen–a component of the natural ECM- has attracted the attention of researchers in the past few years, mainly due to its biocompatibility, biodegradability, low immunogenicity and ability to sustain the optimal wound healing process. Moreover, given its wide range of sources, low costs of extraction, and elevated therapeutic potential, collagen-based dressings represent promising candidates in the field of chronic wound management. However, despite collagen’s advantageous properties, its exclusive use in the crosslinking process leads to hydrogels limited by their weak mechanical properties, inadequate stability, inefficient antibacterial activity, and low biological activity. In order to overcome these challenges, various strategies have been explored, including the blending of collagen with either natural and/or synthetic polymers or bioactive agents with a wide range of antibacterial and biological activities. However, the achievement of a hydrogel system that encompasses good mechanical properties, an efficient antimicrobial effect, and the ability to promote the skin healing process is challenging and requires future research. In addition, the animal models used in the in vivo studies were represented by either mice or rats, meaning that deviations in the experimental results could have occurred due to the fact that the wound healing process of these animals differs from that of human skin. Thus, while most rodent skin can heal wounds by contracting the injury site, the wound healing in humans is achieved through the formation of the granulation tissue and re-epithelialization following injury. With this in mind, more appropriate animal models need to be explored, alongside new approaches that are representative of the human wound healing while using animal models. In the last few years, due to the introduction of animal substitution tests and the extensive research and application of organoids, in vitro constructed skin organoids have been utilized to mimic the human skin environment for more effective research and treatment of skin infections.

While a remarkable advancement has been made in the development of collagen-based wound dressings, there is still considerable room for innovation. Future research should adopt an interdisciplinary approach, combining advances in biomaterials science, nanotechnology, and modern healthcare systems to create personalized and high-performance wound care solutions. By overcoming current limitations and integrating cutting-edge technologies, collagen-based dressings harness the potential to become a key component of next-generation wound care, leading to better patient-reported outcomes and enhanced quality of life.

However, it is also important to recognize that, despite extensive research efforts, only a limited number of collagen-based hydrogel wound care products have received regulatory approval. Bridging the gap between laboratory research and clinical application remains a critical challenge, underscoring the urgent need for translational studies that facilitate the commercialization of promising experimental materials.

## Data Availability

No new data were created or analyzed in this study.
